# Supplementary material for: Regulatory network topology and the genetic architecture of gene expression
Source: bioRxiv. 2025 Aug 12:2025.08.12.669924. Preprint. [Version 1] doi: 10.1101/2025.08.12.669924 (PMC12363847; doi:10.1101/2025.08.12.669924)
Supplement: Supplement 1 [file NIHPP2025.08.12.669924v1-supplement-1.pdf]

bioRxiv preprint doi: https://doi.org/10.1101/2025.08.12.669924; this version posted August 13, 2025. The copyright holder for this preprint (which was not certified by peer review) is the author/funder, who has granted bioRxiv a license to display the preprint in perpetuity. It is made available under aCC-BY 4.0 International license.

598 # 5 Supplementary Information

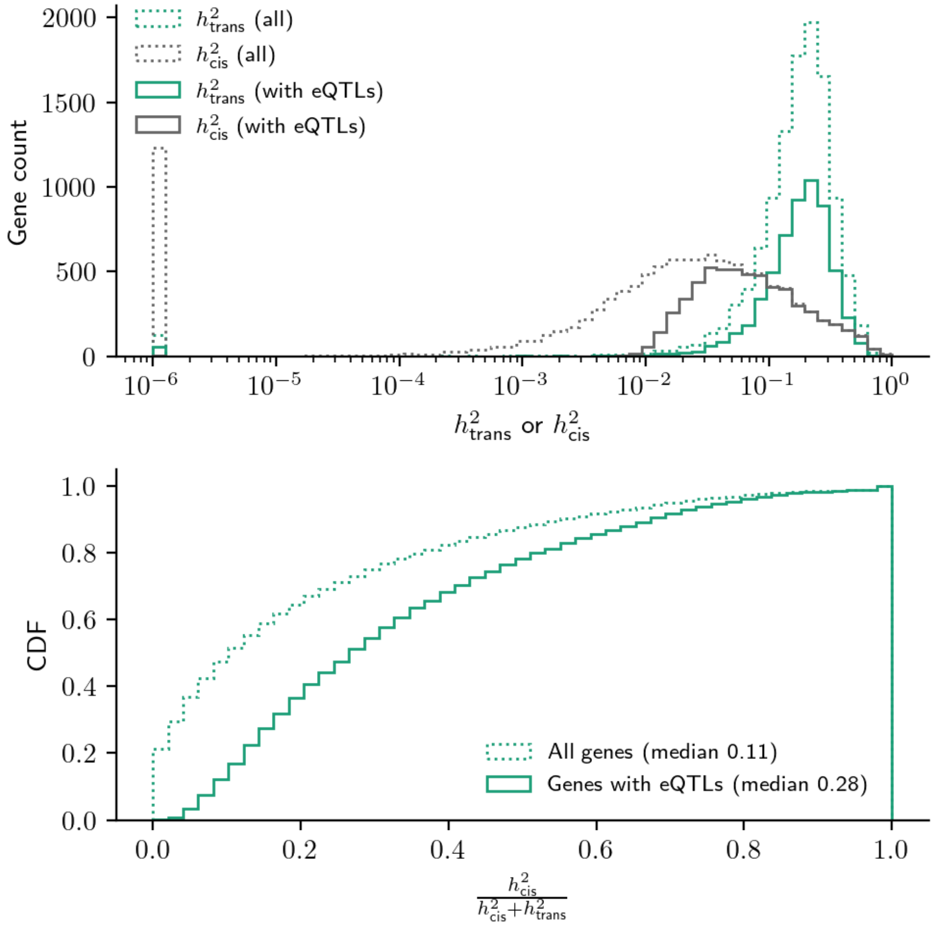


**Figure S1: Distribution of *cis* and *trans* heritability for genes with and without eQTLs**. Data from (Ouwens *et. al.*, 2020). **(A)** Distribution of $h^2_{\text{cis}}$ and $h^2_{\text{trans}}$ for all 11,353 genes with available data from the study (dotted lines) or for all 5,902 genes in the analysis subset from **Fig. 1**, with a detected eQTL (solid lines). **(B)** Distribution of *cis*-heritability fraction from these same gene sets.

bioRxiv preprint doi: https://doi.org/10.1101/2025.08.12.669924; this version posted August 13, 2025. The copyright holder for this preprint (which was not certified by peer review) is the author/funder, who has granted bioRxiv a license to display the preprint in perpetuity. It is made available under aCC-BY 4.0 International license.

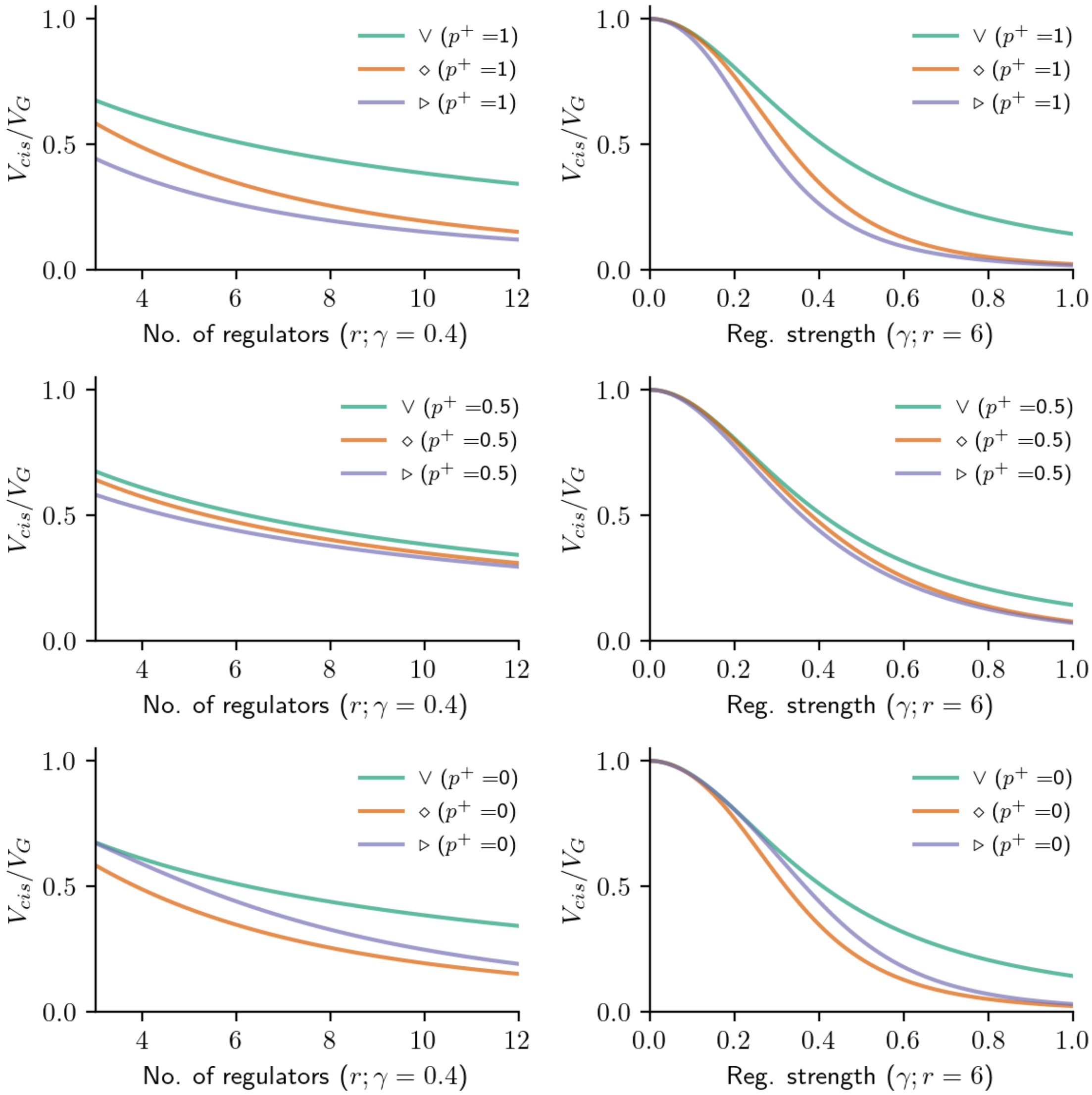


**Figure S2: Effect of motifs with different fractions of activators**. Representative effects of the number of regulators ($r$; left panels) and the strength of regulation ($\gamma$; right panels) on the distribution of *cis*-acting variance for the three motifs in **Fig. 2**. The underlying mathematical equations for vee, triangle, and diamond motifs are as in the main text and corresponding figure — here, the expressions are also stratified by representative fractions of activators $p^+$ (the panels in the top row are exactly as in **Fig. 2**).

bioRxiv preprint doi: https://doi.org/10.1101/2025.08.12.669924; this version posted August 13, 2025. The copyright holder for this preprint (which was not certified by peer review) is the author/funder, who has granted bioRxiv a license to display the preprint in perpetuity. It is made available under aCC-BY 4.0 International license.

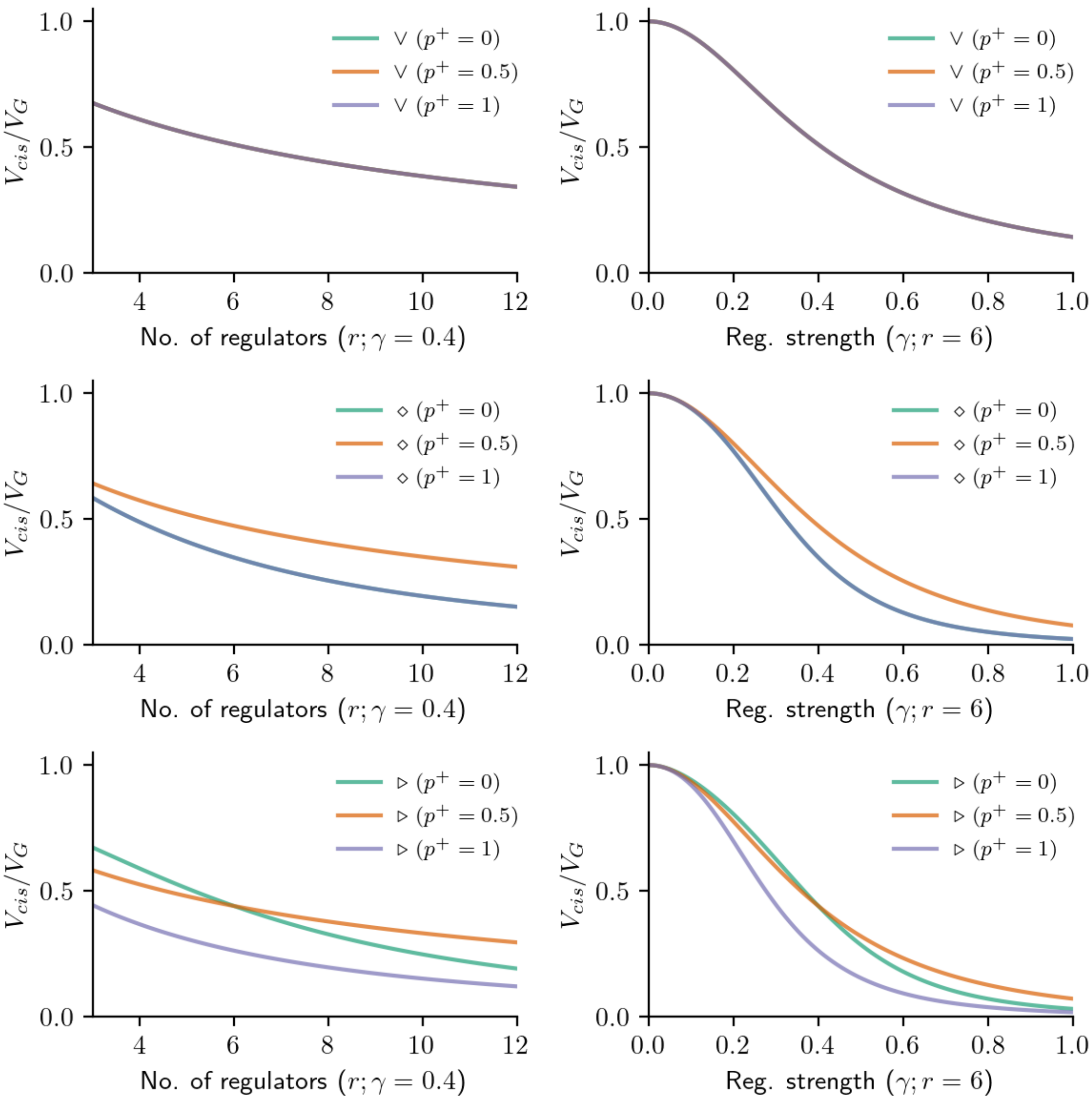


**Figure S3: Effect of regulatory sign on different motifs**. Representative effects of the number of regulators ($r$; left panels) and the strength of regulation ($\gamma$; right panels) on the distribution of *cis*-acting variance for the three motifs in **Fig. 2**. The lines in this plot are the same as in **Fig. S2**, but subpanels here correspond to the separate motifs (vee, diamond, and triangle, in each row) rather than distinct values for the fraction of activators ($p^+$).

bioRxiv preprint doi: https://doi.org/10.1101/2025.08.12.669924; this version posted August 13, 2025. The copyright holder for this preprint (which was not certified by peer review) is the author/funder, who has granted bioRxiv a license to display the preprint in perpetuity. It is made available under aCC-BY 4.0 International license.

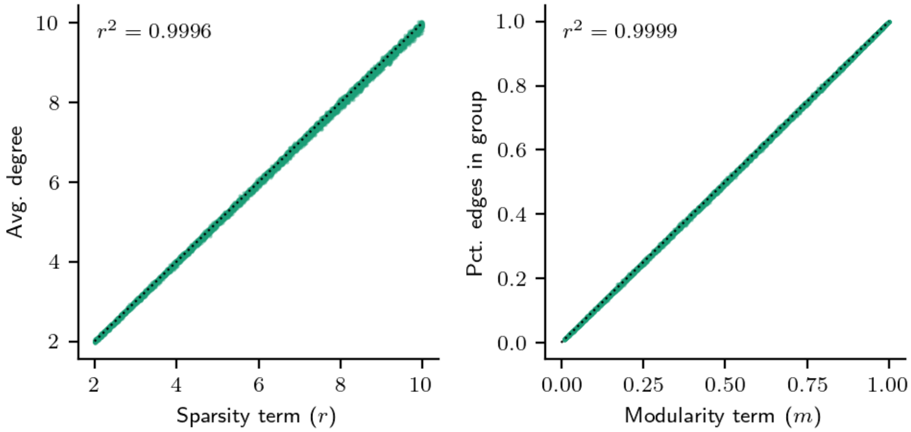


**Figure S4: Parameters of the planted partition model (PPM) control key graph properties**. Values for the sparsity term (*r*; left panel) and modularity term (*m*; right panel) in the 10,000 synthetic GRNs generated using the planted partition model (see **Methods**). The sparsity term *r* is extremely correlated with the average degree in the resulting GRN, and the modularity term *m* is extremely correlated with the resulting fraction of edges in the GRN that are drawn between genes in the same group (rather than genes in separate groups).

bioRxiv preprint doi: https://doi.org/10.1101/2025.08.12.669924; this version posted August 13, 2025. The copyright holder for this preprint (which was not certified by peer review) is the author/funder, who has granted bioRxiv a license to display the preprint in perpetuity. It is made available under aCC-BY 4.0 International license.

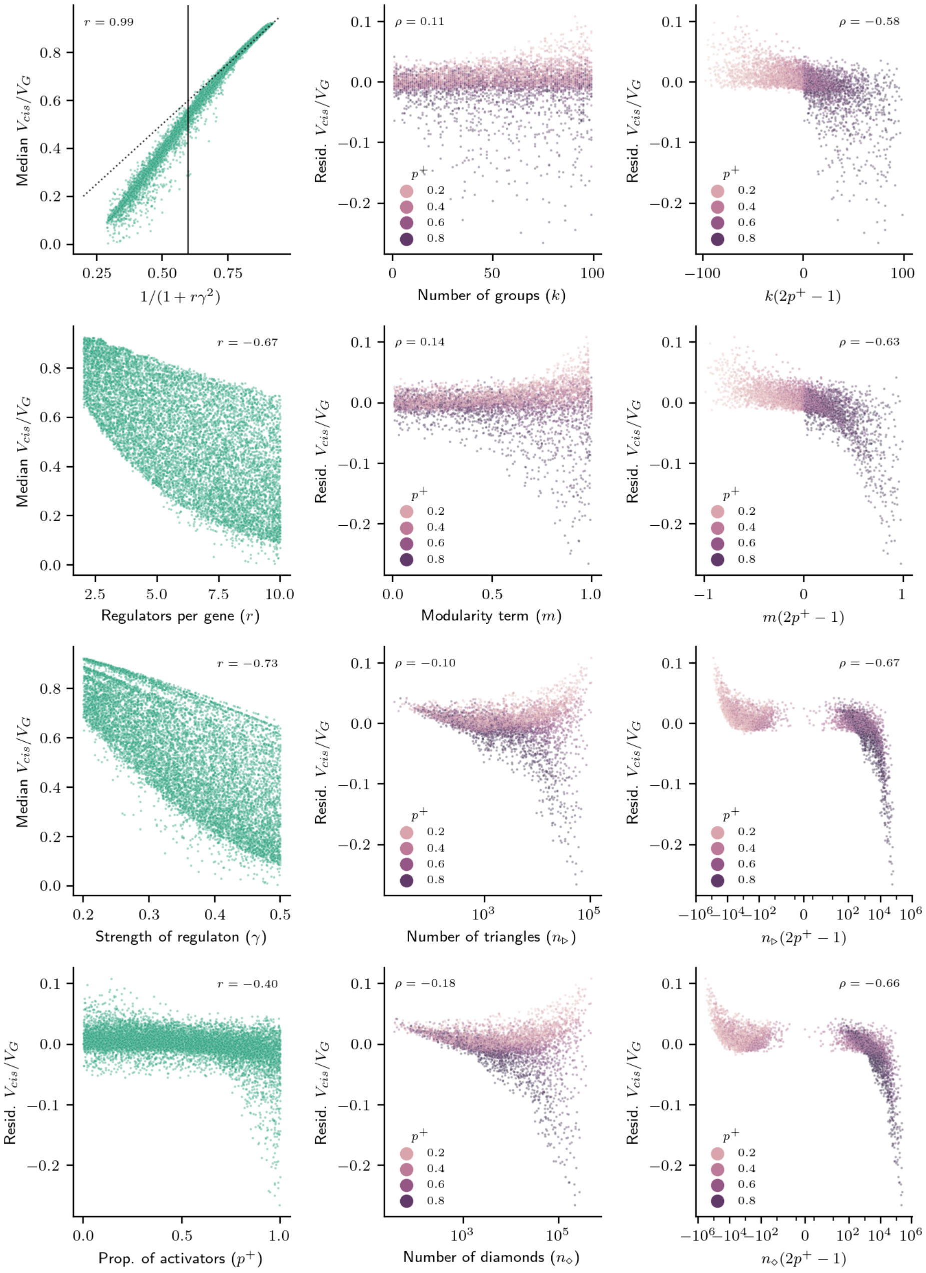


**Figure S5: Parameters of the planted partition model (PPM) affect the distribution of heritability**. Relationships between properties of GRNs produced using the PPM and summaries of the median fraction of *cis*-acting expression variance in the corresponding 10,000 GRNs. Subpanels are annotated by the Pearson ($r$) or Spearman ($\rho$) correlation between the quantities shown on the axes — median $V_{\text{cis}}/V_{\text{G}}$ denotes the untransformed value, while "Resid. $V_{\text{cis}}/V_{\text{G}}$" denotes the residual having regressed out directed effects as $1/(1 + r\gamma^2)$. Networks in the middle and right columns are shown stratified by the fraction of activators $p^+$ and are subsetted to GRNs with lower expected contributions from direct effects ($1/(1 + r\gamma^2) < 0.6$, denoted by a solid vertical line in the top left panel (the dashed line is $y = x$); these are also the networks shown in **Fig. 3**.

bioRxiv preprint doi: https://doi.org/10.1101/2025.08.12.669924; this version posted August 13, 2025. The copyright holder for this preprint (which was not certified by peer review) is the author/funder, who has granted bioRxiv a license to display the preprint in perpetuity. It is made available under aCC-BY 4.0 International license.

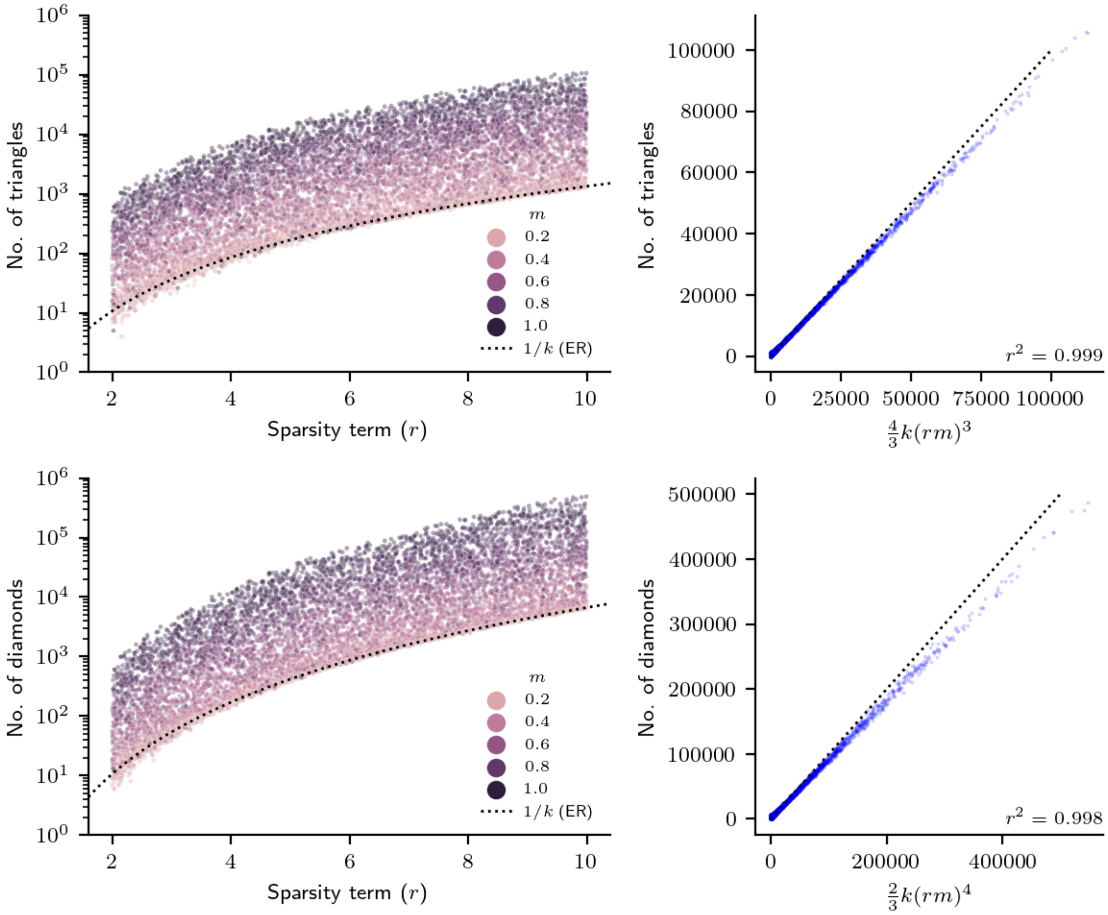


**Figure S6: Parameters of the planted partition model (PPM) affect the number of motifs in the network**. Relationships between the sparsity ($r$) and modularity ($m$) parameters of the PPM and the resulting number of triangle and diamond motifs (defined as diagrammed in **Fig. 2**) in 10,000 PPM GRNs. In both plots, each point is a network — the left panels show the interaction between $r$ and $m$ (note that $m = 1/k$ is the minimum value simulated in the study, and corresponds to the binomial graph); the right panels show the relationship between motif counts and the leading term of a mathematical approximation to the expected number of motifs.

bioRxiv preprint doi: https://doi.org/10.1101/2025.08.12.669924; this version posted August 13, 2025. The copyright holder for this preprint (which was not certified by peer review) is the author/funder, who has granted bioRxiv a license to display the preprint in perpetuity. It is made available under aCC-BY 4.0 International license.

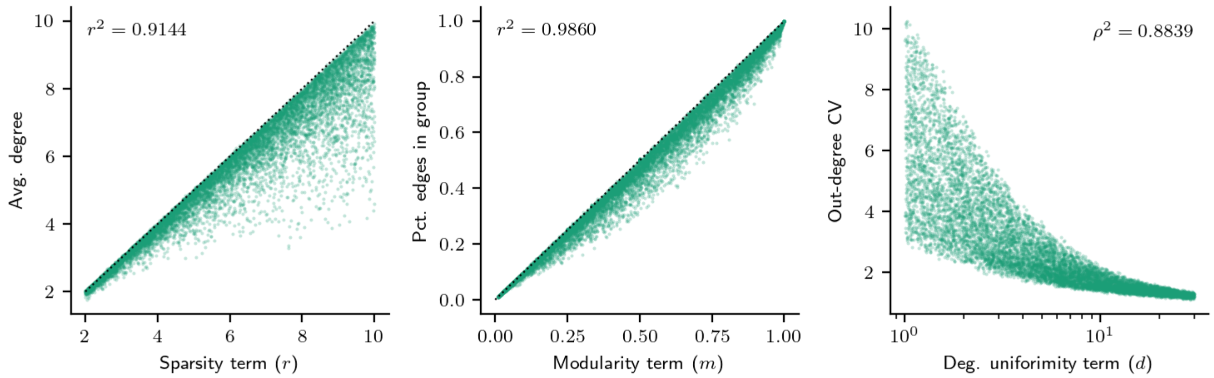


**Figure S7: Parameters of the modular scale-free graph model control key graph properties**. Values for the sparsity term ($r$; left panel) and modularity term ($m$; right panel) in the 10,000 synthetic GRNs generated using the modular scale-free graph generating algorithm (see **Methods**). The sparsity term $r$ is extremely correlated with the average degree in the resulting GRN. The modularity term $m$ is extremely correlated with the resulting fraction of edges in the GRN that are drawn between genes in the same group (rather than genes in separate groups). The degree uniformity term $d$ is strongly correlated with the coefficient of variation (CV) of the out-degree distribution — here, CV is the standard deviation of the degree distribution over its mean, and a larger CV corresponds to a more dispersed distribution.

bioRxiv preprint doi: https://doi.org/10.1101/2025.08.12.669924; this version posted August 13, 2025. The copyright holder for this preprint (which was not certified by peer review) is the author/funder, who has granted bioRxiv a license to display the preprint in perpetuity. It is made available under aCC-BY 4.0 International license.

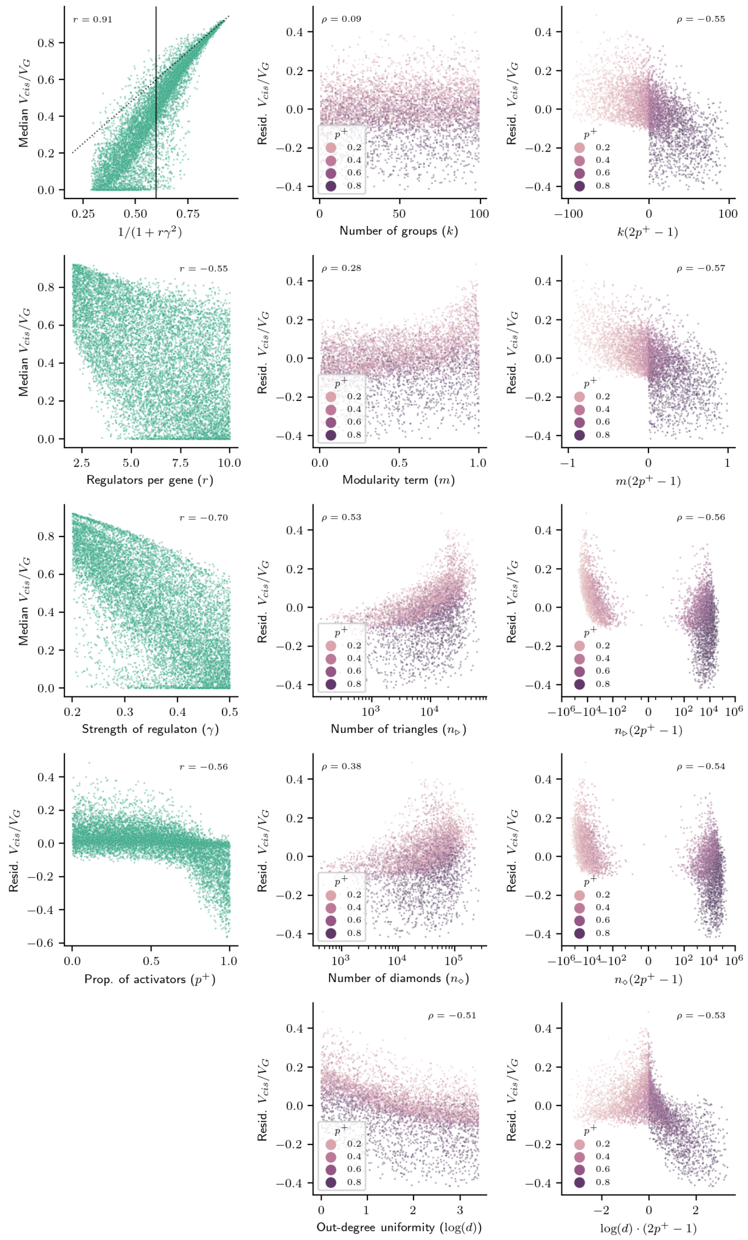

**Figure S8: Parameters of the modular scale-free graph model affect the distribution of heritability**. Relationships between properties of GRNs produced using the modular scale-free graph model and summaries of the median fraction of *cis*-acting expression variance in the corresponding 10,000 GRNs. Subpanels are annotated by the Pearson ($r$) or Spearman ($\rho$) correlation between the quantities shown on the axes — median $V_{\text{cis}}/V_{\text{G}}$ denotes the untransformed value, while "Resid. $V_{\text{cis}}/V_{\text{G}}$" denotes the residual variance after regressing out direct effects as $1/(1 + r\gamma^2)$. Networks in the middle and right columns are shown stratified by the fraction of activators $p^+$ and are subset to GRNs with lower expected contributions from direct effects ($1/(1 + r\gamma^2) < 0.6$, denoted by a solid vertical line in the top left panel (the dashed line is $y = x$); these are also the networks shown in **Fig. 4**.

bioRxiv preprint doi: https://doi.org/10.1101/2025.08.12.669924; this version posted August 13, 2025. The copyright holder for this preprint (which was not certified by peer review) is the author/funder, who has granted bioRxiv a license to display the preprint in perpetuity. It is made available under aCC-BY 4.0 International license.

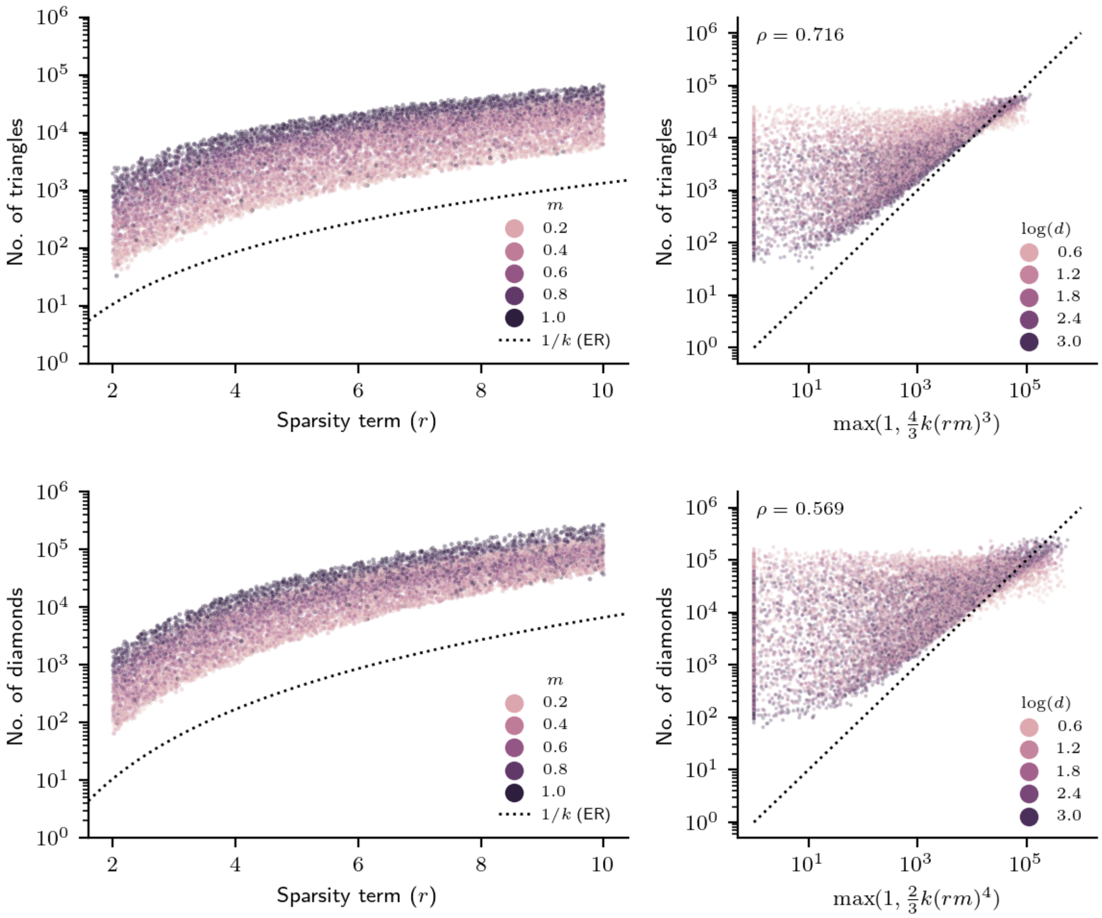


**Figure S9: Parameters of the modular scale-free graph model affect the number of motifs in the network**. Relationships between the sparsity ($r$) and modularity ($m$) parameters of the modular scale-free graph model and the resulting number of triangle and diamond motifs (defined as diagrammed in **Fig. 2**) in 10,000 GRNs. In both plots, each point is a network — the left panels show the interaction between $r$ and $m$ (note that $m = 1/k$ is the minimum value simulated in the study, and corresponds to the value which yields the binomial graph in the PPM); the right panels show the relationship between motif counts and the leading term of a mathematical approximation to the expected number of motifs to the PPM, and that smaller values of $d$ (which introduce hubs into the graph) drive deviations from this expected value.

bioRxiv preprint doi: https://doi.org/10.1101/2025.08.12.669924; this version posted August 13, 2025. The copyright holder for this preprint (which was not certified by peer review) is the author/funder, who has granted bioRxiv a license to display the preprint in perpetuity. It is made available under aCC-BY 4.0 International license.

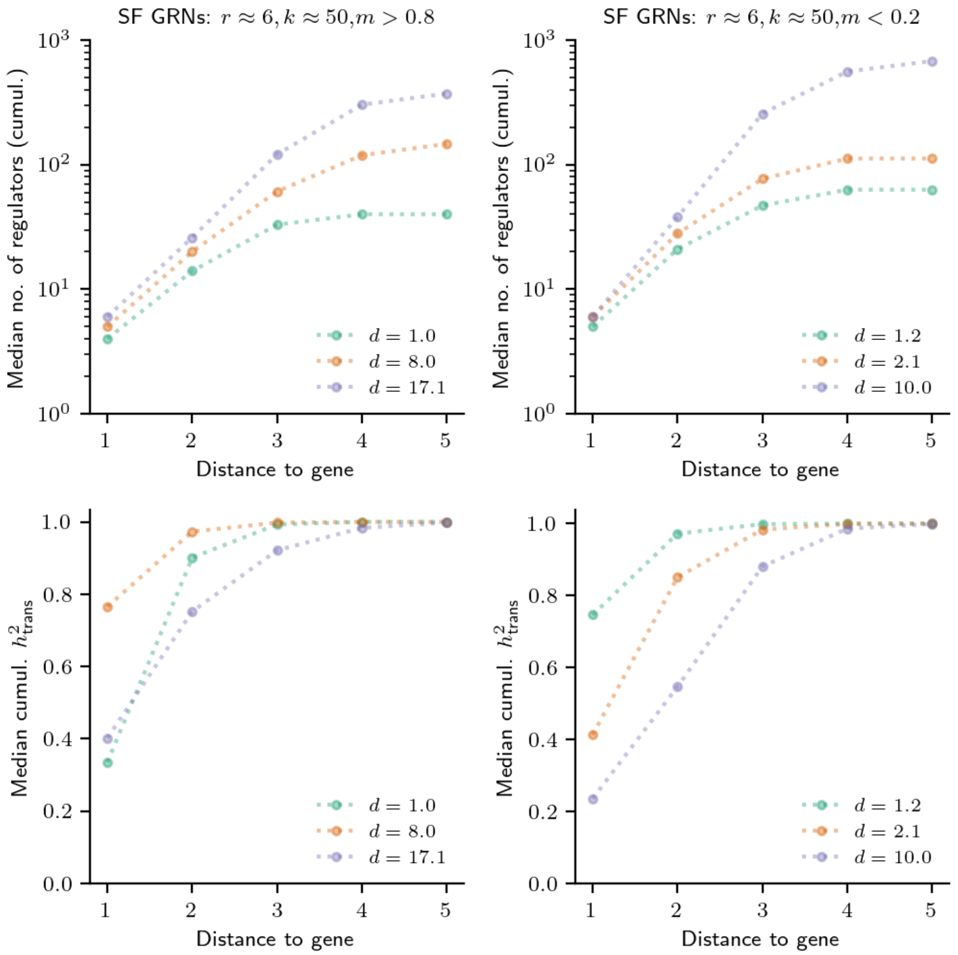


**Figure S10: Degree uniformity alters the distribution of path lengths in the network**. Median number of regulators (top panels) and cumulative *trans*-acting expression variance (bottom panels) in six example modular scale-free GRNs, matched to have similar sparsity ($r \approx 6$) and group structure ($k \approx 50, m > 0.8$ or $m < 0.2$), but vary in other regulatory terms ($d, \gamma, p^+$). Medians are over genes in the middle of the topological sorted order of the DAG (indexes 2000 to 3000). GRNs with regulatory hubs (smaller values of $d$) have fewer distant *trans*-regulators and *trans*-acting heritability is thus typically closer to a given gene in the network.

bioRxiv preprint doi: https://doi.org/10.1101/2025.08.12.669924; this version posted August 13, 2025. The copyright holder for this preprint (which was not certified by peer review) is the author/funder, who has granted bioRxiv a license to display the preprint in perpetuity. It is made available under aCC-BY 4.0 International license.

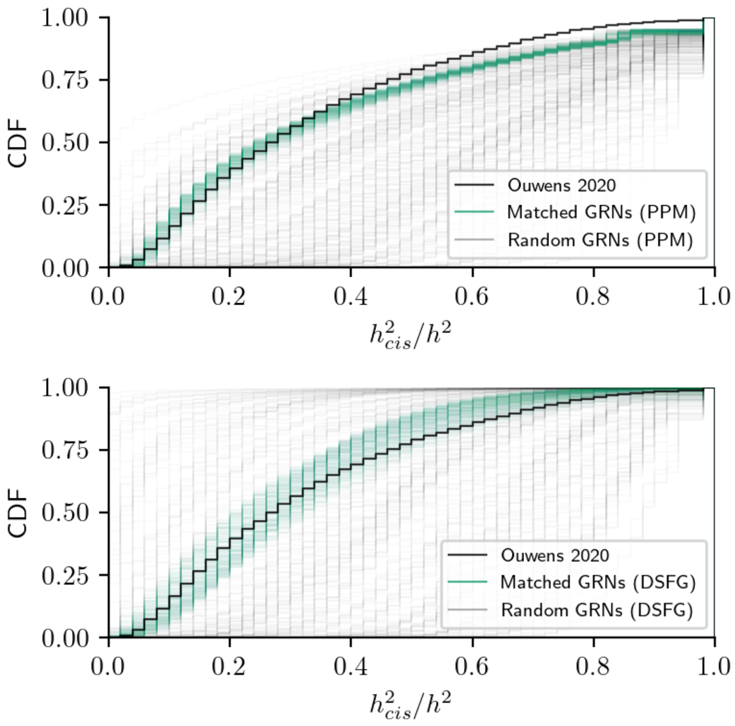


**Figure S11: Distribution of heritability in synthetic GRNs and for whole-blood gene expression**. Distribution of the fraction of *cis*-acting heritability ($h^2_{\text{cis}}/h^2$) from real data (Ouwens *et. al.* 2020) and from synthetic GRNs generated using the planted partition model (PPM; top panel) or the directed scale-free graph generating algorithm (SF; bottom panel). In both plots, teal lines are the 250 GRNs closest to the distribution from data (lowest K-S test statistics; see **Methods**), and grey lines are a random sample of 250 GRNs from the other 9,750 simulated GRNs.

bioRxiv preprint doi: https://doi.org/10.1101/2025.08.12.669924; this version posted August 13, 2025. The copyright holder for this preprint (which was not certified by peer review) is the author/funder, who has granted bioRxiv a license to display the preprint in perpetuity. It is made available under aCC-BY 4.0 International license.

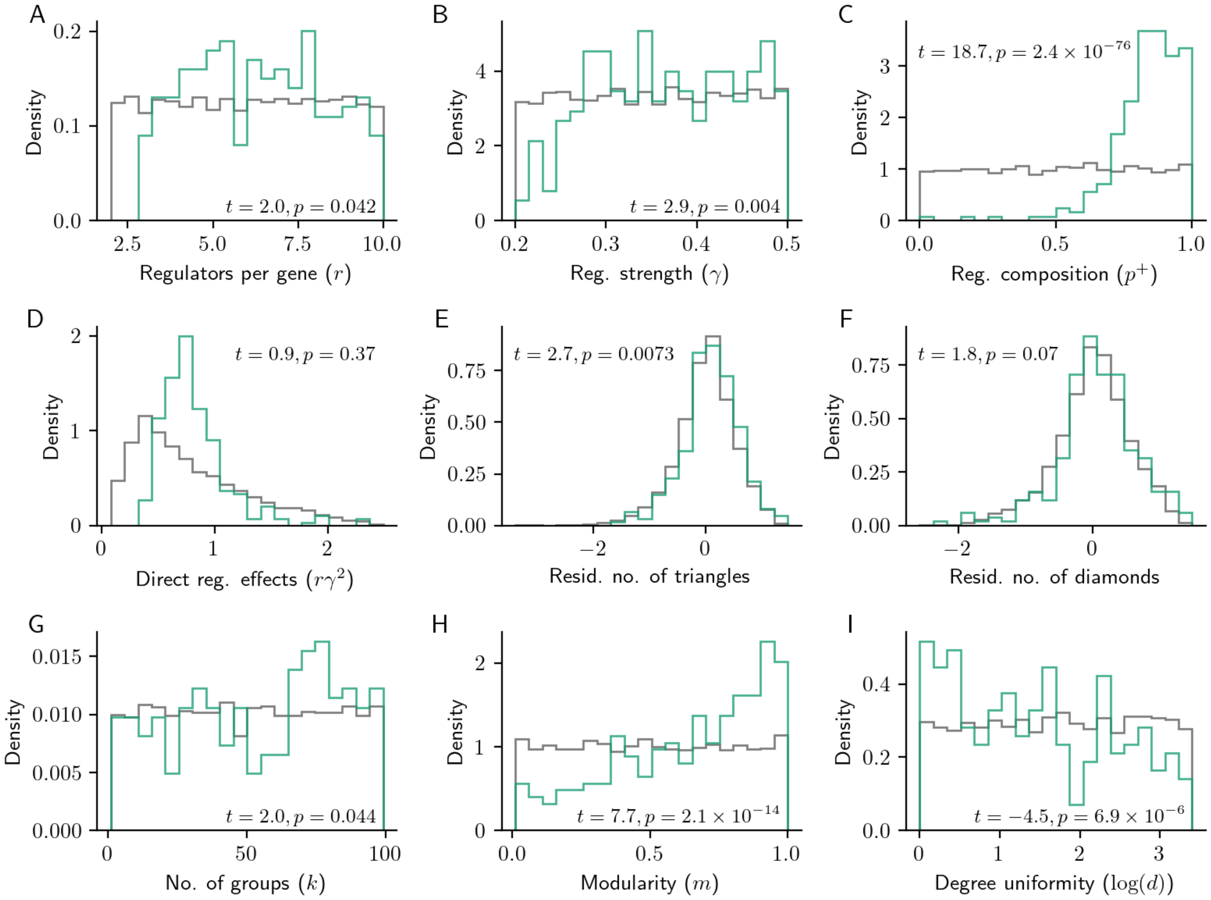


**Figure S12: Properties of synthetic GRNs that best resemble real data**. Additional properties of the 250 GRNs that were best matched to the observed distribution of *cis*-acting heritability fractions (shown in teal, as in **Fig. 5**). Each panel is annotated with results from a two-sample *t*-test for a difference in mean with the remaining 9,750 GRNs (shown in grey).

bioRxiv preprint doi: https://doi.org/10.1101/2025.08.12.669924; this version posted August 13, 2025. The copyright holder for this preprint (which was not certified by peer review) is the author/funder, who has granted bioRxiv a license to display the preprint in perpetuity. It is made available under aCC-BY 4.0 International license.

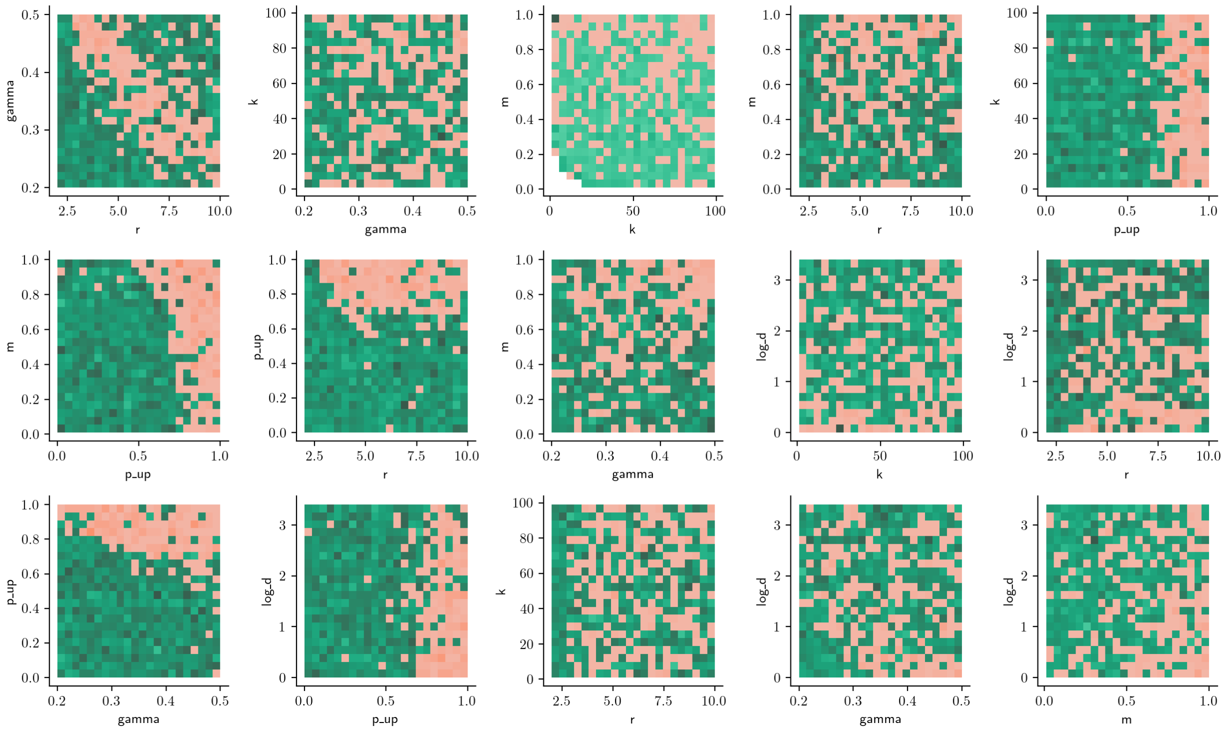


**Figure S13: Interactions between properties of synthetic GRNs that best resemble real expression heritability data**. Pairwise interactions between properties of the 250 GRNs that were best matched to the observed distribution of *cis*-acting heritability fractions. In each subpanel, red denotes an enrichment of matched GRNs in and green denotes depletion.

bioRxiv preprint doi: https://doi.org/10.1101/2025.08.12.669924; this version posted August 13, 2025. The copyright holder for this preprint (which was not certified by peer review) is the author/funder, who has granted bioRxiv a license to display the preprint in perpetuity. It is made available under aCC-BY 4.0 International license.

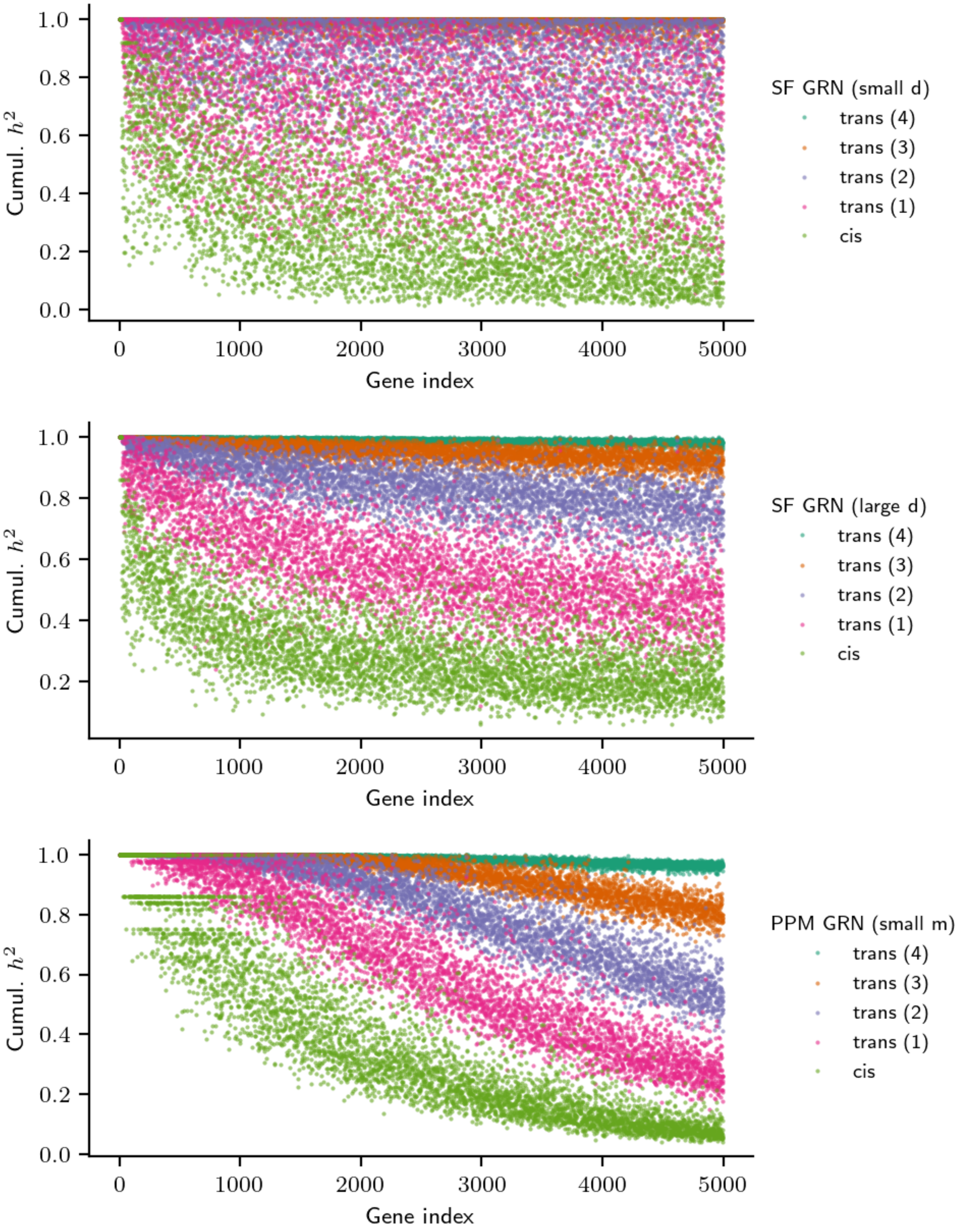


**Figure S14: Gene-level heritability distribution over network distances in three example GRNs**. Cumulative heritability in *cis* and in *trans*, stratified by distance, for all genes in the three example networks from **Fig. 6**.

bioRxiv preprint doi: https://doi.org/10.1101/2025.08.12.669924; this version posted August 13, 2025. The copyright holder for this preprint (which was not certified by peer review) is the author/funder, who has granted bioRxiv a license to display the preprint in perpetuity. It is made available under aCC-BY 4.0 International license.

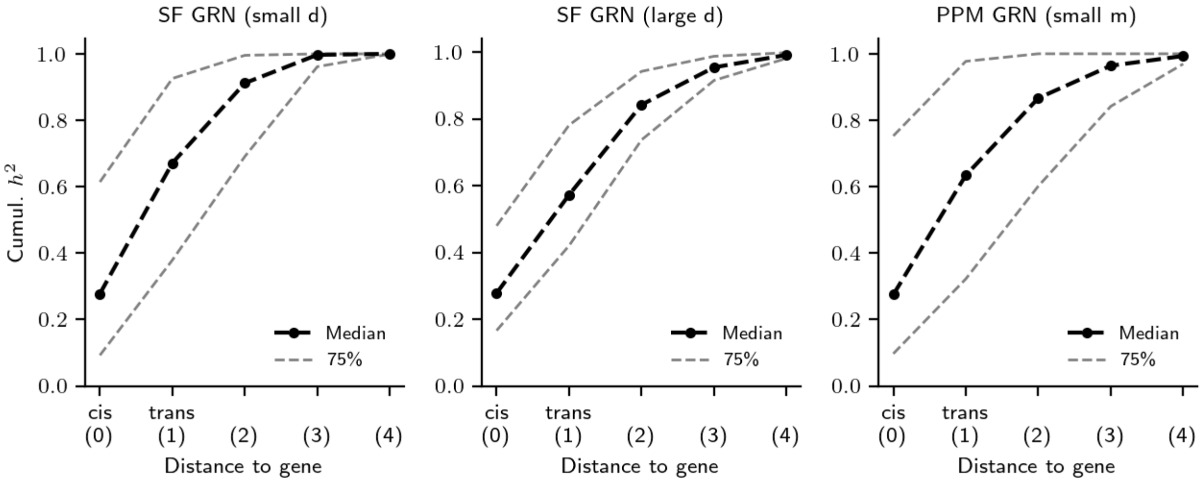


**Figure S15: Cumulative heritability distribution over network distances in three example GRNs**. Cumulative heritability in *cis* and in *trans*, stratified by distance, for the median gene (or quartiles) in the three example networks from **Fig. 6B** — note that the medians and quantiles are computed separately for each tick in the $x$-axis (i.e., the gene with the median *cis*-acting heritability fraction may not be the gene with the median one-hop *trans*-acting heritability fraction).
